# The Frontal Bone Window for Transcranial Doppler Ultrasonography in Critically Ill Patients: Validation of a New Approach in the ICU

**DOI:** 10.1007/s12028-019-00869-3

**Published:** 2019-10-29

**Authors:** Pierre Sentenac, Jonathan Charbit, Camille Maury, Paul Bory, Geoffrey Dagod, Frédéric Greco, Xavier Capdevila, Pierre-François Perrigault

**Affiliations:** 1 Anesthesia and Critical Care Medicine Department, Trauma ICU, Level 1 Regional Trauma Center, Lapeyronie Teaching Hospital, Montpellier University School of Medicine, 34295 Montpellier, France; 2 Anesthesia and Critical Care Medicine Department, Neurological ICU, Gui de Chauliac Teaching Hospital, Montpellier University School of Medicine, 34295 Montpellier, France; 3 PhyMedExp, Unité 1046, Institut National de la Santé et de la Recherche Médicale, Centre National de la Recherche Scientifique, University of Montpellier, 34295 Montpellier, France; 4 Institut des Neurosciences de Montpellier (INM), Unité 1051, Institut National de la Santé et de la Recherche Médicale, University of Montpellier, 34091 Montpellier, France; 5 Anesthesia and Critical Care Medicine Department, Heart and Lung center, Arnaud de Villeneuve Teaching Hospital, Montpellier University School of Medicine, 371 avenue du Doyen Gaston Giraud, 34295 Montpellier, France

**Keywords:** Transcranial Doppler ultrasonography (D017585), Anterior cerebral artery (D020771), Cerebral vasospasm (D020301), Neurophysiological monitoring (D064926), Reliability and validity (D015203), Frontal acoustic bone window

## Abstract

**Background and Objective:**

The temporal bone window (TBW) for transcranial Doppler (TCD) often fails to insonate the anterior cerebral artery (ACA). The frontal bone window (FBW) has never been evaluated in intensive care units (ICU). The main objective was to determine the ability of the FBW to assess ACA velocities in critically ill patients.

**Methods:**

A prospective study was conducted in two ICUs of the Montpellier University Hospital (France), between November 2014 and September 2016. Adult patients admitted to ICU for brain injury, with a Glasgow Coma Scale score ≤ 13, were enrolled within 3 days after admission. A first TCD examination was carried out bilaterally through the TBW and FBW by an intensivist expert in TCD, repeated by the same examiner, and 15 min later by an intensivist certified in TCD, designated as non-expert, blinded. The success of the FBW examinations was defined by the ability to measure the ACA velocities. Intra- and interobserver agreements were analyzed according to the Bland and Altman method.

**Results:**

A total of 147 patients were analyzed. The FBW succeeded in insonating the ACA in 66 patients [45%, CI (37–53)], 45 bilaterally and 21 unilaterally. For 16 patients (11%), the FBW was the only way to measure ACA velocities. By combining the two techniques, the ACA success rate increased from 62% CI (54–70) to 73% CI (65–79) (*P *= 0.05). Intra- and interobserver mean biases and 95% limits of agreement for ACA systolic velocity measurements through the FBW were 1 (− 33 to 35) and 2 (− 34 to 38) cm s^−1^, respectively. For paired TBW and FBW measures of ACA velocities, mean biases (± SD) for ACA systolic, and mean and diastolic velocities were relatively close to zero, but negatives (− 7 ± 33, − 2 ± 19, − 1 ± 15 cm s^−1^, respectively), highlighting that ACA velocities were lower with the FBW (A2 segment) than TBW (A1 segment). The correlation coefficient for ACA systolic velocities measured by the FBW and TBW was *R *= 0.47, CI (0.28–0.62). No risk factors for failure of the FBW were identified.

**Conclusions:**

In ICU, the FBW was able to insonate the ACA in 45% of patients admitted for brain injury, without the use of contrast agents. The FBW could improve the detection of ACA vasospasms.

**Electronic supplementary material:**

The online version of this article (10.1007/s12028-019-00869-3) contains supplementary material, which is available to authorized users.

## Introduction

Transcranial Doppler ultrasonography (TCD) is a crucial monitoring tool in neurocritical care units [1–3]. TCD is particularly useful in patients suffering from aneurysmal subarachnoid hemorrhage (SAH) [4–6], traumatic brain injury (TBI) [7–9], or cerebral stroke [10, 11]. TCD is reputed to be a low-cost and readily repeatable diagnostic imaging test, offering a noninvasive real-time monitoring of cerebral circulation at bedside, which suits perfectly for the intensive care unit (ICU) setting [1–3].

In order to insonate basal cerebral arteries and Willis polygon, the ultrasound beams have to penetrate the skull through a proper acoustic bone window. The temporal bone window (TBW) described by Aaslid et al. [12] in 1982 has become the standard approach for TCD examination in adults. However, the conventional TBW has two main limits. First, velocity measurement is not feasible in approximately 10–30% of patients by TBW due to a lack of echogenicity [2, 13, 14]. Second, the TBW is most often inadequate to insonate the anterior cerebral artery (ACA), especially the A2 segment, because of unfavorable angle of insonation [2, 23]. A segmental disease of the ACA, such as a vasospasm occurring in case of SAH, will be consequently undetectable using TBW [6, 15–18]. At times, patients receive delayed catheter-based therapies due to the fact that the ACA could not be insonated.

First described in neuropediatric and neuroradiology, the frontal bone window (FBW) is a poorly known alternative approach for TCD [19–23]. By positioning the probe above the supraorbital arcade, the FBW allows to measure the ACA velocities with low-angle correction. Previous studies in non-critically ill patients showed that the FBW allowed to assess cerebral artery velocities when the TBW failed [23]. Nevertheless, these observations were not established in critically ill patients, who are more likely to develop cerebral vasospasms.

The main purpose of the present study was to determine the ability of the FBW to assess ACA velocities in patients admitted to ICU for brain injury. Secondary goals were to define risk factors for failure, intra- and interobserver agreement, and correlation between TBW and FBW measures of ACA velocities.

## Materials and Methods

### Study Design

A prospective study was conducted in the two critical care units of the Montpellier University Hospital (France), the trauma ICU and the neurological ICU. The study has been performed in accordance with the ethical standards as laid down in the 1964 Declaration of Helsinki. The institutional review board waived the need for informed consent (IDRCB-2014A0143641). The registration number was NCT02832895.

### Eligibility Criteria

Patients 18 years of age or older, admitted to ICU for brain injury (i.e., TBI; SAH; intracranial hematoma; cerebral stroke; post-cardiac arrest syndrome; encephalitis; or craniotomy for brain tumor) with an initial Glasgow Coma Scale score less than or equal to 13, were eligible for the present study. Patients were enrolled within the first 3 days after admission, once they were clinically stable. Patients with clinical suspicion of brain death, or refusing to participate (or refusal of the legally authorized representative), were excluded from analysis.

### Data Collection

Clinical characteristics and biological parameters upon inclusion were noted, such as body temperature, mean arterial pressure, glycemia, natremia, hemoglobin level, and arterial blood gas values. The hospital length of stay and survival on hospital discharge were collected.

### TCD Protocol

According to a standardized protocol, a first TCD examination was carried out bilaterally by the TBW and FBW in all studied patients by an intensivist expert in TCD imaging (with more than 10 years of experience in TCD imaging in ICU, designated as referent for TCD in the center, and teaching the new FBW). Subsequently, the same examiner performed 15 min later a second TCD examination through the FBW. Afterward, a third TCD examination was performed 15 min later through the FBW by a second examiner. The second examiner, blinded to the results of the expert, was an intensivist certified in ultrasound imaging and trained to the new technique (i.e., more than 30 TCD examinations through the FBW), designated as non-expert. All TCD examinations were achieved in clinically stable patients, postured in supine position with 15-30° head up. The maximal duration of each examination was arbitrarily fixed to 10 min. No clinical or therapeutic interventions were allowed during the study procedure.

TCD examinations were performed using ultrasound machines Vivid-i™ or Vivid-q™ (GE Healthcare^®^, Chicago, USA), connected to a 3S-RS^®^ (GE Healthcare^®^) adult probe (1.7-4 MHz). The settings of ultrasound machines are as follows: 2D mode, emission frequency of 2.5 MHz; color mode, emission frequency of 1.8 MHz, gain of 15 dB, and scale of 2 kHz. Ultrasound contrast agents were not allowed. Once the targeted blood vessel was insonated with the two-dimensional color-coded image, velocities were measured using angle-corrected pulsed wave Doppler. According to guidelines [2], Doppler tracing lasting at least 10 cardiac cycles was recorded after a 30-s stabilized period and the cycle with the highest systolic velocity was studied. Peak systolic velocity, end-diastolic velocity, and mean velocity (respectively, SV, DV, and MV, expressed in cm s^−1^) were measured, and the pulsatility index (corresponding to [SV–DV]/MV) was determined. Depth of measurement (expressed in cm) and angle correction (expressed in degrees) were also collected.

**Fig. 1:**
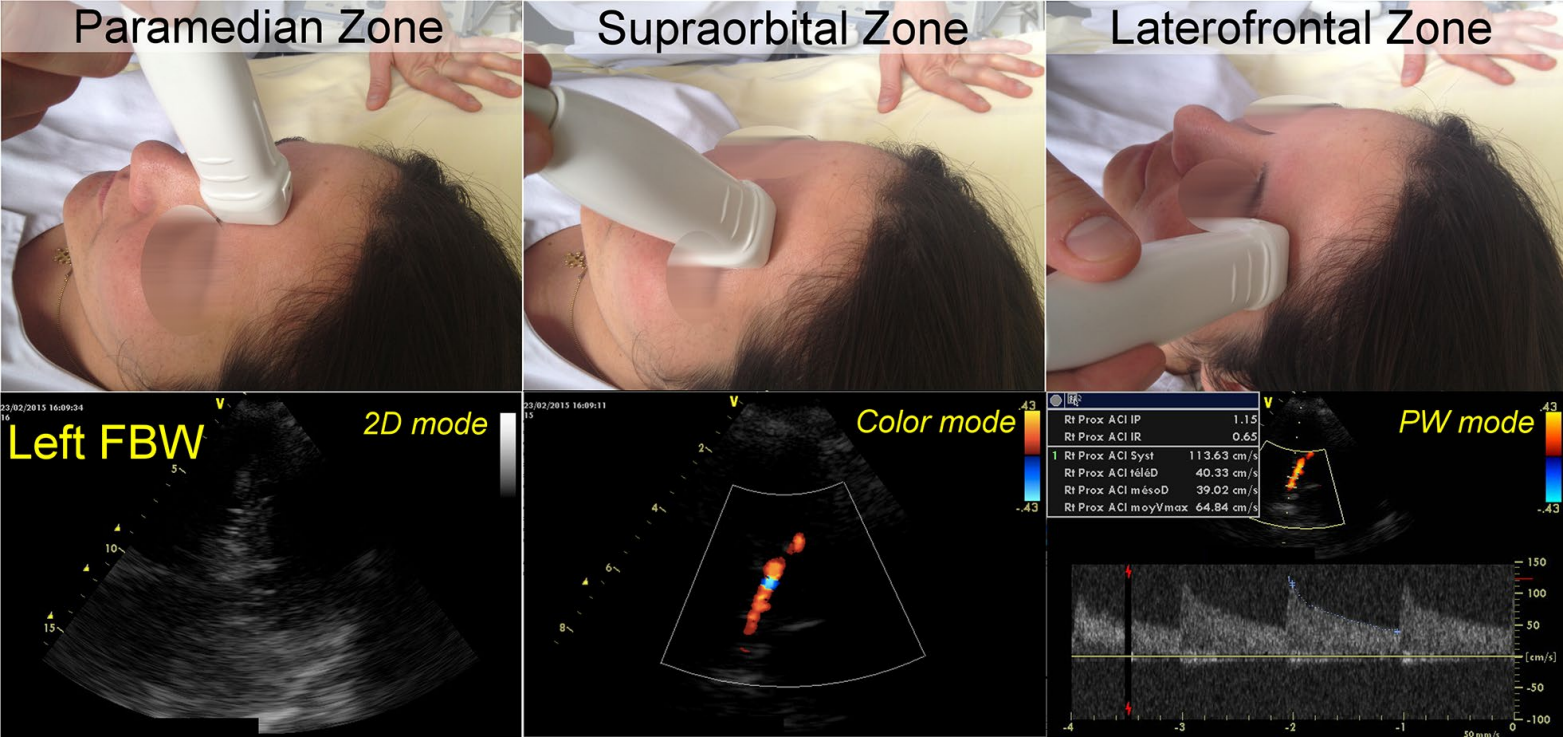
Description of the FBW technique for TCD ultrasonography. The transducer is positioned vertically at the paramedian frontal zone (index mark at the top), rotated 90° outward and shifted horizontally (index mark laterally) to the supraorbital zone at the top of the orbital arcade, up to the laterofrontal zone. Visualization of the brain parenchyma in B-mode (two-dimensional mode), insonation of the A2 segment of the ACA in color mode, and blood flow velocity measurements using the pulsed Doppler (PW) mode. [*ACA* anterior cerebral artery, *PW* pulsed waved Doppler]

The FBW examinations were conducted as previously described [19–23]: The transducer was positioned vertically at the paramedian frontal zone (index mark at the top), rotated 90° outward, and moved horizontally (index mark laterally) to the supraorbital zone at the top of the orbital arcade, up to the laterofrontal zone (Fig. 1 and Supplementary video). Only the A2 segments of the ACA were recorded by the FBW, while only the A1 segments were measured by the TBW. If ACA was insonated with multiple approaches, the one with maximal velocities was reported.

### Main study Endpoints

The success of the TBW and FBW examinations was defined by the ability to measure the ACA velocities on the first TCD examination. Patients were thus categorized into two groups: the FBW success group (unilateral or bilateral success of FBW examination) or the FBW failure group (bilateral failure of FBW examination). The diagnostic contribution of the FBW was expressed as the increase in the proportion of patients with a TCD success for the ACA, from the TBW examination to the combination of TBW + FBW.

### Statistical Analysis

The study population was first described, and characteristics of the groups FBW failure and FBW success were compared. Qualitative variables were expressed by number (percentage) and tested using the Chi-squared test. For quantitative variables, the normality of distribution was tested using Shapiro–Wilk test and expressed as mean (SD) or median (IQR). Unpaired quantitative data were compared using Student’s t test or Mann–Whitney test. Paired quantitative data were compared using paired Student’s t test or Wilcoxon test.

The TBW and FBW success was described as percentages with 95% confidence intervals (CI). As recent literature reported a FBW success rate of about 40% in non-critically ill patients [23], a sample size of 150 subjects would have approximately 80% power to determine a success rate arbitrarily estimated to 40%, using a 95% CI with a ± 8% accuracy, considering an alpha risk of 0.05. Risk factors for FBW failure were investigated. Intra- and interobserver concordance was evaluated by the kappa coefficient. Agreement between ACA measurements was subsequently studied according to the Bland and Altman method [24], expressed as mean bias with 95% limits of agreement (LOA). The statistical relationship between A1 (TBW) and A2 (FBW) velocities was expressed using the Pearson’s correlation coefficient (*r*).

Calculated *P* values less than or equal to 0.05 were considered statistically significant for all two-sided tests. Statistical analysis was performed using Prism 6™ software (GraphPad^®^, La Jolla, USA).

## Results

### Study Population

Between November 2014 and September 2016, 152 patients were included in the present study (Fig. S1). Five were secondarily excluded (two for consent withdrawal and three for missing data). Therefore, a total of 147 patients were analyzed, 60 (41%) in the trauma ICU and 87 (59%) in the neurological ICU.

The cohort consisted of 96 men (65%), with mean age 51 ± 19 years (Table 1). The main causes of ICU admission were TBI (27%) and SAH (25%). Eleven patients (8%) had frontotemporal decompressive craniectomy. At the time of the study, clinical and biological parameters were stabilized. The mean length of stay was 21 ± 20 days, simplified acute physiology score (SAPS II) was 43 ± 17, and mortality rate on day 28 was 27%.

**Table 1:** Study population (*n* = 147 patients)

Characteristics	All patients (*n* = 147)
Demographic data	
Age, mean ± SD, y	51 ± 19
Gender, no. of men (%)/women (%)	96 (65)/51 (35)
Body mass index, mean ± SD, kg/m2	26 ± 5
Severity	
Initial GCS score, mean ± SD	7 ± 4
SAPS score, mean ± SD	43 ± 17
Length of stay in ICU, mean ± SD, *d*	21 ± 20
Mortality on day 28, No. (%)	40 (27%)
Pathology, no. (%)	
Traumatic brain injury	39 (27)
Subarachnoid hemorrhage	37 (25)
Spontaneous intracranial hematoma	18 (12)
Ischemic stroke	17 (12)
Post-cardiac arrest syndrome	15 (10)
Metabolic encephalopathy	14 (9)
Brain tumor	7 (5)
Decompressive craniectomy, no. (%)	11 (7)
Inclusion day, no. (%)	
Day 1	35 (24)
Day 2	48 (33)
Day 3	64 (43)
Sedation at inclusion day, no. (%)	
No sedative drugs	29 (20)
Midazolam + Sufentanil	100 (68)
Other drugs	18 (12)
Clinical parameters and laboratory values at inclusion, mean ± SD	
Body temperature, °C	36.9 ± 1.1
Mean arterial pressure, mmHg	85 ± 13
Glycemia, g/L	1.3 ± 0.3
Arterial SO2, %	98 ± 2
Arterial PCO2, mmHg	37 ± 6
Arterial pH	7.41 ± 0.07
Natremia, mmol/L	141 ± 4
Hemoglobin, g/L	117 ± 20

_*GCS* Glasgow Coma Scale, *PCO2* partial pressure of carbon dioxide, *SAPS* simplified acute physiology score, *SD* standard deviation, *SO2* oxygen saturation_

### TBW and FBW Success Rates

Among the studied population, the TBW and FBW success rates were 62% (95% CI 54–70%) and 45% (95% CI 37–53%), respectively. The FBW success rate did not differ significantly between the two participating units: 48% (95% CI 35–60%) in the trauma ICU vs. 41% (95% CI 30–51%) in the neurological ICU (*P *= 0.41).

### Diagnostic Contribution of the FBW

**Fig. 2:**
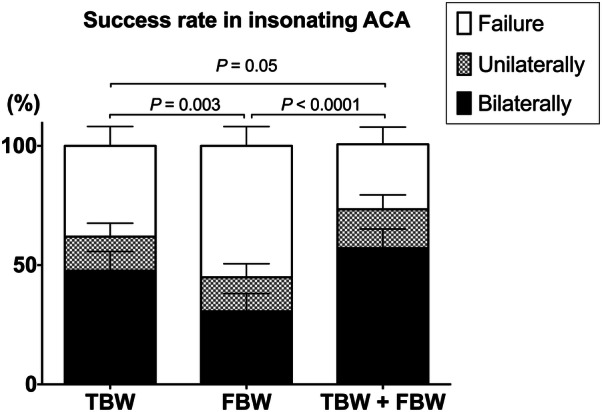
Success rates in insonating the ACA by the TBW, FBW and the combination of both techniques. The success is the ability to insonate the ACA, uni- or bilaterally (stacked percentage bar plot and upper range of the 95% confidence interval). One-way ANOVA, *P* values for Tukey’s multiple comparisons tests. [*ACA* anterior cerebral artery, *FBW* frontal bone window, *TBW* temporal bone window]

In our population, the TBW did not provide bilateral assessment of ACA velocities in 77 patients (52%), including 56 (38%) with bilateral TBW failure (Fig. S1). Among them, the FBW was a success in 25 (29%). Moreover, for 16 of these patients, the FBW was the only way to measure ACA velocities. The ACA success rate increased therefore from 62% (95% CI 54–70%) using only the TBW to 73% (95% CI 65–79%) using the combination TBW + FBW (*P *= 0.05) (Fig. 2). Thus, the diagnostic contribution of the FBW for ACA was estimated to a + 11% increase of the ACA insonation rate.

### Risk Factors for the FBW Failure

The univariate analyses did not reveal a variable significantly associated with an increased risk of FBW failure. The relative risks (RR) of the most commonly known risk factors, an age over 60 and the female gender, were 1.2 (95% CI 0.9–1.5) and 1.2 (95% CI 0.9–1.6), respectively. Craniectomy was not significantly associated with a lower FBW failure rate (RR = 0.6, 95% CI 0.3–1.4). The FBW success rate did not differ significantly between non-craniectomized patients (*n* = 136) and the entire cohort (Table S2 and Figure S2).

### Agreement Between TBW and FBW Measures

All data derived from the first TCD examination are presented in Table S1. Notably, the ACA systolic velocities were lower on average when measured by the FBW (A2 segment) than TBW (A1), 79 ± 31 vs. 93 ± 38 cm s^−1^, respectively (*P *= 0.04), as well as pulsatility index. The other parameters did not differ significantly between the two windows.

Agreement between paired TBW and FBW measures of ACA velocities is presented in Table 2. Mean biases (± SD) for ACA systolic and mean and diastolic velocities were relatively close to zero, but negatives (− 7 ± 33, − 2 ± 19, − 1 ± 15 cm s^−1^, respectively), confirming that ACA velocities were lower using the FBW (A2 segment).

**Table 2:** Intra- and interobserver agreements for the FBW, and correlation between TBW and FBW measures

	FBW		FBW *vs.* TBW
ACA measurements	Intraobserver agreement	Interobserver agreement	Agreement between A2 (FBW) and A1 (TBW) measures
Systolic velocity (cm s^−1^)			
Mean bias ± SD	1 ± 17	2 ± 18	-7 ± 33
Range (95% LOA)	68 (− 33 to 35)	72 (− 34 to 38)	131 (− 73 to 58)
*R* (95% CI)	0.84 (0.77–0.88)	0.64 (0.47–0.77)	0.47 (0.28–0.62)
*R*^2^	0.70	0.41	0.22
Mean velocity (cm s^−1^)			
Mean bias ± SD	1 ± 9	3 ± 14	-2 ± 19
Range (95% LOA)	35 (− 17 to 18)	54 (− 24 to 30)	75 (− 39 to 36)
*R* (95% CI)	0.90 (0.85–0.93)	0.70 (0.57–0.79)	0.42 (0.23–0.59)
*R*^2^	0.80	0.49	0.18
Diastolic velocity (cm s^−1^)			
Mean bias ± SD	0 ± 6	-2 ± 9	-1 ± 15
Range (95% LOA)	22 (− 11 to 11)	34 (− 19 to 15)	60 (− 31 to 29)
*R* (95% CI)	0.87 (0.82–0.91)	0.69 (0.56–0.78)	0.17 (-0.06–0.39)
*R*^2^	0.76	0.48	0.03
Pulsatility index			
Mean bias ± SD	0 ± 0.18	0 ± 0.22	-0.07 ± 0.28
Range (95% LOA)	0.72 (− 0.36 to 0.36)	0.84 (− 0.42 to 0.42)	1.10 (− 0.62 to 0.48)
*R* (95% CI)	0.78 (0.70–0.85)	0.71 (0.59–0.80)	0.53 (0.35–0.67)
*R*^2^	0.62	0.50	0.28

*ACA* anterior cerebral artery, *A1* segment A1 of the ACA, *A2* segment A2 of the ACA, *CI* confidence interval; *FBW* frontal bone window, *LOA* limits of agreement, *R* Pearson’s correlation coefficient; *R*^2^ determination coefficient, *SD* standard deviation, *TBW* temporal bone window, *TCD* transcranial Doppler

### Intra- and Interobserver Agreement of FBW Measures

In 100% of cases, the second TCD examination was a success when the first TCD examination succeeded, leading to an intraobserver kappa coefficient of 1.00 (95% CI 0.89–1.11). Besides, the third TCD examination performed by blinded examiners was a success in 90% of cases when the first TCD examination succeeded, leading to an interobserver kappa coefficient of 0.80 (95% CI 0.69–0.91).

**Fig. 3:**
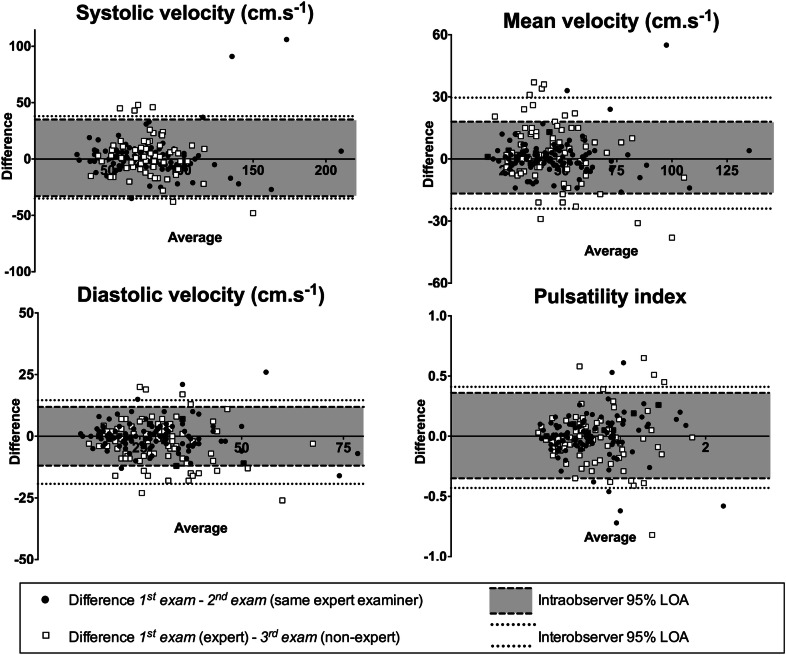
Intra- and interobserver agreement for measurements made by the FBW. Bland and Altman plots representing intra- and interobserver 95% LOA for ACA systolic, mean, diastolic velocities and pulsatility index measured by the FBW. [*ACA* anterior cerebral artery, *FBW* frontal bone window, *LOA* limits of agreement]

The assessment of intra- and interobserver agreement for ACA velocities and pulsatility index measurements is presented in Table 2. Intra- and interobserver agreements for ACA systolic and mean and diastolic velocities were optimal, with biases close to zero and a limited dispersion (1 ± 17, 1 ± 9, 0 ± 6, and 2 ± 18, 3 ± 14, − 2 ± 9 cm s^−1^, respectively). Likewise, a strong correlation between measures was found. The corresponding Bland and Altman plots are presented in Fig. 3. It is noteworthy that the intraobserver and interobserver agreements for systolic velocity were comparable in our series, meant by similar 95% LOA (− 33 to 35 cm s^−1^ and − 34 to 38 cm s^−1^, respectively).

### Technical Considerations

Technically, the ACA was more frequently insonated in the paramedian and supraorbital areas (39 and 37% of measurements, respectively), while the laterofrontal zone was the less performing (24%) (Table S3). Duration of FBW examinations was 5 ± 3 min on average. The angle correction for measuring ACA velocities tended to be lower through FBW than TBW, 9 ± 14° vs. 22 ± 20°, without reaching significance (*P *= 0.27).

## Discussion

The FBW for TCD was evaluated for the first time in 147 critically ill patients, the largest cohort studied to date. Our population consisted of 65% men, mean age of 51 years, and TBI and SAH were the leading causes of ICU admission. In the present population, the FBW succeeded in insonating the ACA in about one in two patients. The reproducibility was excellent; intra- and interobserver mean biases for ACA velocities were close to zero with limited dispersion. The ACA detection rate increased from 62 to 73% using the combination TBW + FBW, an absolute gain of 11%.

### A Crucial Need for ACA Evaluation

In patients with SAH, cerebral velocities are monitored regularly by TCD to detect vasospasm [2–5]. To be efficient, this monitoring requires a specific segmental evaluation of cerebral velocities. The TBW, standard approach for TCD, does not allow in most cases to insonate the A2 segment of the ACA, which is perpendicular to the probe. A segmental vasospasm of A2 will be consequently undetectable by the TBW [15–18], which decreases the chances of receiving appropriate catheter-based therapies, highlighting the need for complementary TCD approaches. Therefore, by adding the FBW approach, treatable lesions of the ACA could be identified which would have gone unnoticed with an isolated TBW approach.

### The FBW: A Complementary TCD Technique for Insonating the ACA

First described in a pediatric population [20], the FBW provided ACA measures in 80% of cases. Nevertheless, it is well known that echogenicity in adults is much lower. In a cohort of 163 adults with stroke [23], the FBW success rate was 37% without the use of contrast agents. In line with these results, the FBW success rate was 45% in our cohort. It has been previously shown that the FBW success rate is improved by intravenous contrast agents. Stolz et al. [21] described a success rate of 73% in 75 healthy volunteers. Three years later, the same authors published a detection rate of 86% in 40 control patients with echocontrast enhancement [22]. The use of echocontrast agents could certainly improve the success rate of the FBW in ICU, but further investigations are needed.

### Risk Factors for FBW Failure

It is well known that elderly people and female gender are risk factors for failure of the TBW examination [2, 14]. The same risk factors for FBW failure were previously described. Stolz et al. [21] reported that echogenicity of the FBW decreased in women and elderly people, with a strong decrease of ACA insonation rate, 85–22%, when comparing patients younger than 40 and those older than 60 years. In our study, older age and female gender trended to be associated with FBW failure, without reaching significance. Our success rates were 55% in patients younger than 40 and 45% over 60. It is likely that the improvement of echogenicity by echocontrast agents is more marked in young than elderly people. Craniectomized patients tended to have a higher success rate than the non-craniectomized. But this subgroup was small (*n* = 11) and did not influence the results of the entire cohort (Table S2 and Figure S2). The impact of the frontal bone thickness and calcium content on the FBW echogenicity rate and 2D-imaging quality could be investigated in future studies. Ethnicity was not investigated to comply with the French legislation.

### Agreement Between the FBW and TBW Measures

The agreement between ACA velocities measured by the two techniques has already been studied, showing contrasting results. Stolz et al. [22] reported that ACA systolic velocity was higher when measured by the FBW than TBW in healthy patients, 92 ± 23 and 78 ± 16 cm s^−1^. Our study showed contrasting results, with ACA systolic velocities lower when measured by the FBW (A2 segment) than TBW (A1). To our understanding, this difference is mainly explainable by the fact that A2 is more distal along the arterial tree. Furthermore, the Doppler angle correction was higher for the A1 segment (TBW) than A2 (FBW), which could have led to measurement biases.

### Reproducibility of the FBW Technique

To our knowledge, the reproducibility of the FBW had never been evaluated before. In our study, intra- and interobserver agreements of ACA systolic, diastolic, mean velocities, and pulsatility index measurements were excellent with biases close to zero and limited dispersions. Remarkably, intra- and interobserver limits of agreement for systolic velocity measurements were similar, meaning that the expert and non-expert examiners found closely the same values for ACA systolic velocities. For mean and diastolic velocities, larger interobserver LOA was noted, but without clinical relevance. A plausible explanation for this finding is that a small absolute error of ± 5 cm s^−1^ could have induced a larger relative error for low velocities (diastolic and mean velocity) than high velocities (systolic velocity). Nevertheless, in both healthy patients and SAH, Staalsø et al. [25] found larger intra- and interobserver LOA for MCA mean velocity measured by the TBW than those we observed for ACA by the FBW. Reproducibility of angle correction was not investigated.

### The Added Value of the FBW: Improving the ACA Detection Rate

In the study of Yoshimura et al. [23], the combined application of TBW and FBW improved the detection rates of A1 segment from 46 to 59%, and A2 segment from 7 to 44% compared to the TBW alone. The limit of the TBW examination was highlighted in our study, since the TBW was unable to provide bilateral ACA measurements in 52% of patients, which is consistent with previous observations [23]. The FBW provided measurements in about a third of these patients, representing one in six patients of the entire cohort. Thus, by combining the FBW with the standard TBW evaluation, the ACA success rate increased significantly from 62 to 73% of patients. In order to investigate the additional clinical benefit of the FBW, it will be important to study whether abnormal findings are identified by either or both methods, and whether either improves diagnostic certainty or results in changed management. Until then, the importance of an 11% increase in yield for identifying the ACA (any segment) will remain uncertain.

### Clinical Perspectives

In clinical practice, the intended use of the FBW examination could be a complement to the TBW examination, not a replacement, as is the case with other alternative approaches [26–28]. Even if focused on different segments, the FBW might be important as a way to make sure at least some of the ACA is identified, or maybe more important as a way to study the A2, which is rarely identified by TBW. Therefore, the new FBW examination could have decisive implications for the bedside monitoring in critically ill patients at risk of vasospasm, such as patients with SAH, in whom a segmental monitoring of cerebral blood flow velocities is crucial.

### Limitations of the Study

First, our TBW success rate for insonating the ACA was lower than expected despite the concordance with the findings of Yoshimura et al. [23]. There is a lack of published literature about TCD success rates in ICU, varying from one center to another. Success rates would probably be higher if examinations were performed by expert sonographers rather than intensivists, or with the use of contrast agents. We assume that FBW is a technically difficult test that should be performed by experts, or at least certified intensivists. Even if our results could certainly be transposed to ICU with high-performance ultrasound machines, the interest of FBW is probably more limited in centers performing TCD without imaging assistance. The B-mode also allowed angle correction which is a parameter that must be taken into account when comparing our results with previous literature.

Second, the diagnostic accuracy of the FBW examination was not easy to determine using our study design. There is no gold standard for assessing flow velocities in the A2 segment of the ACA. Test results were not categorized as either positive or negative, so the familiar diagnostic accuracy statistics such as sensitivity, specificity, and predictive values were not estimated [29, 30]. Determining a test positivity cutoff was not possible in our heterogeneous study population. The diagnostic gain of the FBW has to be investigated in further studies in larger cohorts of ICU patients with SAH.

## Conclusions

The FBW was able to insonate the ACA in 45% of patients admitted to ICU for brain injury, without the use of contrast agents. The reproducibility was excellent. Combining the TBW with the FBW significantly enhanced the insonation rate of the ACA when compared to the TBW alone. The FBW for TCD could have major implications for clinical practice, mainly improve detection of cerebral vasospasms.

## Electronic supplementary material

Below is the link to the electronic supplementary material.

Supplementary material 1 (MP4 26130 kb)

Supplementary material 2 (DOCX 211 kb)
