# Supplementary material for: The Frontal Bone Window for Transcranial Doppler Ultrasonography in Critically Ill Patients: Validation of a New Approach in the ICU
Source: Neurocrit Care. 2019 Oct 29;33(1):115–23. doi: 10.1007/s12028-019-00869-3 (PMC7392931; doi:10.1007/s12028-019-00869-3)
Supplement: Supplementary file 2 — Supplementary material 2 (DOCX 211 kb) [file 12028_2019_869_MOESM2_ESM.docx]

SUPPLEMENTARY DATA

**THE FRONTAL BONE WINDOW FOR TRANSCRANIAL DOPPLER ULTRASONOGRAPHY IN CRITICALLY-ILL PATIENTS: VALIDATION OF A NEW APPROACH IN ICU**

**Supplementary Figures**

**Fig. S1 Flow diagram of study participants and results from the *first TCD exam* performed by the TBW and FBW**. The success of the TBW and FBW exams were defined as the ability to insonate the ACA, unilaterally or bilaterally. Underlined in bold red (*), patients with missing values of ACA velocities after the TBW exam, for whom the FBW provided the corresponding measurements. [ACA = anterior cerebral artery ; FBW = frontal bone window ; TBW = temporal bone window ; TCD = transcranial Doppler]


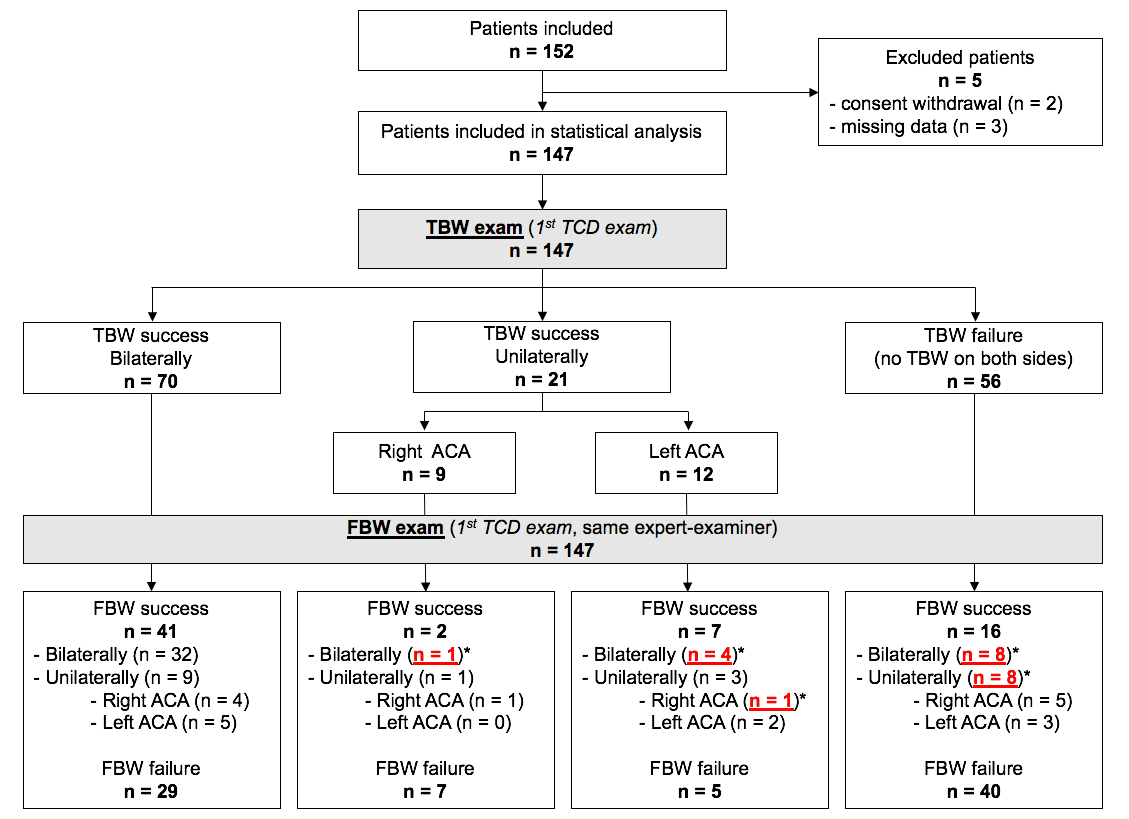


**Figure S2. Success rates of the TBW (*panel A*) and FBW (*panel B*) in the entire cohort population (n = 147), in craniectomized patients (n = 11) and non-craniectomized patients (n = 136).** The success is the ability to insonate the ACA, uni or bilaterally (stacked percentage bar plot and upper range of the 95% confidence interval). One-way ANOVA, Tukey’s multiple comparisons tests. [ACA = anterior cerebral artery ; FBW = frontal bone window ; TBW = temporal bone window]


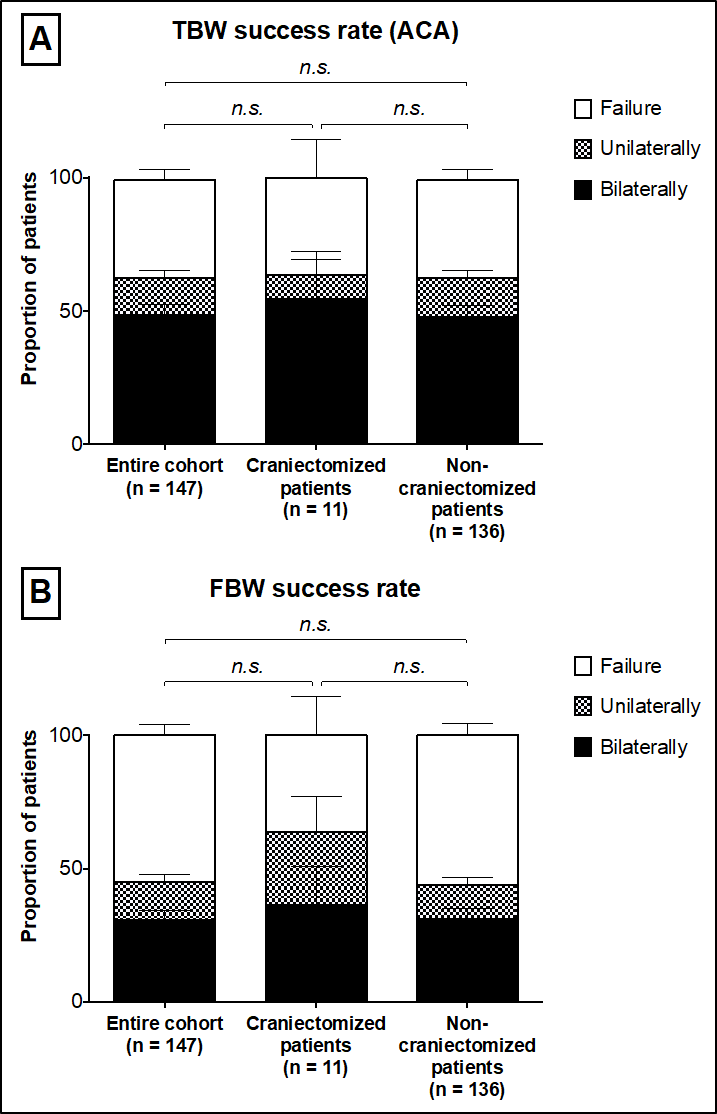


**Supplementary Tables**

**Table S1**. **TCD parameters measured by the FBW and TBW (results of the *first* TCD exam).**

|  | **FBW** |  | **TBW** |  |
| --- | --- | --- | --- | --- |
| **Insonated artery** | **ACA (A2)** | **ACA (A1)** | **MCA** | **PCA** |
| **No. measures / No. vessels  (%)** | 112 / 294  (38) | 162 / 294  (55) | 256 / 294  (87) | 168 / 294  (57) |
| **Velocities, mean ± SD, cm.s^-1^** |  |  |  |  |
| Systolic velocity  (range) | 79 ± 31 (30—226) | 93 ± 38 (29—215) | 103 ± 40 (37—393) | 79 ± 36 (26—259) |
| Mean velocity  (range) | 47 ± 20 (17—137) | 52 ± 22 (18—143) | 59 ± 28 (16—337) | 47 ± 25 (11—168) |
| Diastolic velocity  (range) | 28 ± 12 (11—75) | 31 ± 14 (9—94) | 36 ± 22 (5—286) | 29 ± 16 (5—106) |
| **Calculated indexes, mean ± SD** |  |  |  |  |
| Pulsatility index | 1.1 ± 0.3 | 1.2 ± 0.3 | 1.2 ± 0.4 | 1.1 ± 0.4 |
| Resistance index | 0.64 ± 0.1 | 0.65 ± 0.09 | 0.66 ± 0.1 | 0.63 ± 0.1 |
| **Technical information, mean ± SD** |  |  |  |  |
| Angle correction, ° | 9 ± 14 | 22 ± 16 | 8 ± 16 | 11 ± 18 |
| Depth of measurement, cm | 5.5 ± 0.9 | 6.1 ± 0.8 | 5.2 ± 0.7 | 5.9 ± 0.7 |
| Study duration, min | 5 ± 3 |  | 10 ± 5 |  |
| TCD: transcranial Doppler ; FBW: frontal bone window ; TBW: temporal bone window ; ACA: anterior cerebral artery ; MCA: middle cerebral artery ; PCA: posterior cerebral artery. | | | | |

**Table S2. Characteristics of craniectomized patients and results from the first TCD exam in this subgroup**. A success is coded by 1 and a failure by 0. [A = Trauma center ; C = Neurological ICU ; M = male ; F = female ; TBI = traumatic brain injury ; SAH = subarachnoid hemorrhage]

| **Participant number** | **Center** | **Sex** | **Age** | **Pathology** | **Side of craniectomy** | **Right TBW** | **Left TBW** | **Right FBW** | **Left FBW** | **FBW success or failure** |
| --- | --- | --- | --- | --- | --- | --- | --- | --- | --- | --- |
| #023 | A | M | 73 | Intracranial hemorrhage | Right | 1 | 1 | 0 | 0 | Failure |
| #096 | A | M | 37 | TBI | Right | 0 | 0 | 0 | 0 | Failure |
| #103 | A | M | 49 | TBI | Left | 0 | 0 | 0 | 1 | Success (unilaterally) |
| #037 | C | M | 27 | TBI | Left | 1 | 1 | 1 | 1 | Success (bilaterally) |
| #047 | C | M | 62 | SAH | Right | 0 | 1 | 0 | 1 | Success (unilaterally) |
| #049 | C | M | 23 | TBI | Left | 0 | 0 | 0 | 0 | Failure |
| #059 | C | F | 64 | Stroke | Left | 1 | 1 | 1 | 1 | Success (bilaterally) |
| #104 | C | F | 45 | SAH | Right | 1 | 1 | 1 | 1 | Success (bilaterally) |
| #114 | C | F | 45 | Stroke | Left | 1 | 1 | 0 | 1 | Success (unilaterally) |
| #119 | C | M | 64 | TBI | Left | 1 | 1 | 0 | 0 | Failure |
| #127 | C | M | 41 | SAH | Right | 0 | 0 | 1 | 1 | Success (bilaterally) |

**Table S3. Angle correction values from the first TCD exam**. [ACA = anterior cerebral artery ; TBW = temporal bone window ; FBW = frontal bone window ; TCD = transcranial Doppler]

| **TCD window** | **Angle correction (°)**  **mean ± SD (range)** |
| --- | --- |
| **TBW (A1, ACA)** | 22 ± 16 (0—68) |
| **FBW (A2, ACA)** | 9 ± 14 (0—60) |
| - Paramedian approach (39%) | 5 ± 17 (0—31) |
| - Supraorbital approach (37%) | 10 ± 9 (0—60) |
| - Laterofrontal approach (24%) | 15 ± 17 (0—50) |

**Supplementary Videos:**

**Supplementary Video Legend**: The Frontal Bone Window for Transcranial Doppler: description of the technique, and illustrations from clinical cases.
